# The Growth Attainment, Hematological, Iron Status and Inflammatory Profile of Guatemalan Juvenile End-Stage Renal Disease Patients

**DOI:** 10.1371/journal.pone.0140062

**Published:** 2015-10-07

**Authors:** Juliana Casimiro de Almeida, Randall Lou-Meda, Marion Olbert, Markus Seifert, Günter Weiss, Erwin T. Wiegerinck, Dorine W. Swinkels, Noel W. Solomons, Klaus Schümann

**Affiliations:** 1 Center for Studies of Sensory Impairment, Aging and Metabolism (CeSSIAM), Guatemala City, Guatemala; 2 MBR Optical Systems, Wuppertal, Germany; 3 Fundación para el Niño Enfermo Renal (FUNDANIER), Roosevelt Hospital, Guatemala City, Guatemala; 4 Department of Internal Medicine VI, Medical University of Innsbruck, Innsbruck, Austria; 5 Department of Laboratory Medicine, Radboud University Medical Center, Nijmegen, the Netherlands; 6 Hepcidinanalysis.com at the Department of Laboratory Medicine of the Radboud University Nijmegen Medical Center, Nijmegen, the Netherlands; 7 Molecular Nutrition Unit, ZIEL, Research Center for Nutrition and Food Science, Technische Universität München, Freising, Germany; University of Sao Paulo Medical School, BRAZIL

## Abstract

**Background:**

Stunting, anemia and inflammation are frequently observed in children with end-stage renal disease (ESRD).

**Objectives:**

To assess anthropometric, hematological and inflammatory data and to study their potential interrelationship in Guatemalan juveniles undergoing hemodialysis (HD) and peritoneal dialysis (PD).

**Methods:**

54 juveniles 7–20 years of age were recruited in FUNDANIER, Guatemala City: 27 on HD and 27 PD. Hemoglobin, serum iron, transferrin, serum transferrin receptor (sTfR), serum ferritin, transferrin saturation and iron-binding capacity, white blood cell count (WBC), erythrocyte sedimentation rate (ESR), C-reactive protein (CRP), as well as IL-6, IL-1 and TNF-α, weight and height were determined by standard methods. Hepcidin–25 (Hep-25) was assessed by weak cation exchange time-of-flight mass-spectrometry.

**Results:**

92% and 55% of HD and PD children, respectively, were stunted and 95% and 85% were anemic. Among iron status biomarkers, serum ferritin was massively increased and significantly higher in the HD group compared to the PD group. Hep-25 was also greatly elevated in both groups. 41% of HD patients showed increments in three or more inflammatory biomarkers, while it was 2 or less in all PD subjects.

**Conclusions:**

The degree of stunting, the prevalence and severity of anemia in Guatemalan juvenile ESRD far exceed the national statistics for this low-income Central American country. Ferritin and Hep-25 concentrations were elevated, with the latter to an extraordinary magnitude. Additional biomarkers of inflammation not directly related to iron status were elevated as well. The role of both disease- and environment-related factors in combination best explains the magnitude of the biomarker abnormalities.

## Introduction

Guatemala is a country in Central America, bordered to the north and to the west by Mexico, to the northeast by Belize, to the east by Honduras and to the southeast by El Salvador. The capital, Guatemala City, is located 1,500 m above sea level. Although Guatemala is the biggest economy in Central America, it is among Latin American countries with the highest levels of inequality, with poverty indicators among the highest in the region [1]. Guatemala ranks 125^th^ out of 187 countries on the Human Development Index (HDI) in 2014 [2]. Guatemala is considered a lower middle income developing economy with a 2013 gross domestic product (GDP) of $54 billion [1]. Over 50% of Guatemala’s people live below the national poverty line and 15% in extreme poverty [3].

Preventable diseases result in death, since access to quality health care is scarce. Infant mortality rate is high, although it has been declining (47 per 1000 live births in 2000 to 25 per 1000 in 2012) [2].

Guatemala has the highest prevalence of under-five stunting in the Latin American region at 49.8% in the 2008–2009 survey [4]. It also has a legacy of anemia, with pregnant women and children under five having prevalences of 22% and 38%, respectively [5]. Recurrent diarrheal disease [6], intestinal parasites infestation [7,8], and giardiasis [9,10], are among the environmental factors leading to inflammatory stress. Rural homes have interior fireplaces and poor ventilation with consequent indoor smoke contamination [11,12] and questions have been raised as to the microbiological quality of the urban water supply [13,14]. A background of poor growth, anemia and inflammation-inducing factors is present within the contemporary Guatemalan society.

The diverse etiological factors for end-stage renal disease (ESRD) are common to industrialized societies and to developing countries alike. The resources and technology for extracorporeal dialysis are rare in developing countries. Experiences have been reported from the Middle East [15,16], South America [17,18], and Africa [19,20]. Nevertheless, reports on juvenile kidney disease progressing to ESRD in developing countries are scarce. Malnutrition constitutes an intertwined concern of juvenile ESRD. Conservative means to preserve failing renal function often involve restriction of protein-rich foods, a source of nitrogenous waste. Such dietary restriction, however, contributes to weight loss and loss of muscle and visceral mass [21]. In children who have yet to achieve their adult stature, malnutrition is not only reflected by a low body mass index (BMI), but also by decreased height. Stunting is a common consequence of childhood renal disease [22,23].

Hematological abnormalities are common in ESRD. They are associated with loss of renal parenchymal tissue, the source of erythropoietin (EPO), which upregulates erythropoiesis [24] and with hyporesponsiveness to erythropoiesis stimulation agents [25]. EPO resistance along with chronic inflammation in ESRD create the classical conditions for “anemia of chronic disease” mediated by increased hepcidin levels [26].

Systemic inflammation is often reflected by increased circulating C-reactive protein (CRP) levels. Along with Interleukin-6 (IL-6) [27] and tumor necrosis factor alpha (TNF-α) [28], it is a consistent predictor of mortality in patients with ESRD [27–29]. An important pathway to fatal outcomes is through the elevated incidence of cardiovascular diseases [30]. This concern of inflammatory-induced vascular damage is especially valid in children, who will experience years of exposure [30].

The Foundation for Children with Kidney Diseases (FUNDANIER) is based in the Department of Pediatrics of the state-run Roosevelt Hospital in Guatemala City. It has five hemodialysis (HD) stations allowing for 15 sessions daily in three shifts, and a stable population of 30 patients on every-other-day HD. Home peritoneal dialysis (PD), in the form of Continuous Ambulatory Peritoneal Dialysis (CAPD), is the other therapy offered, and it has managed up to 90 PD patients at a time.

The treatment for ESRD in FUNDANIER is free of charge. However, the hospital does not have the means to provide the required medicine to the patients. Therefore it falls onto the families to obtain the medications. Sixty-eight per cent of the patients in FUNDANIER come from the metropolitan and central regions of Guatemala [31,32]. Once the rural areas are reached, the number of children in need of RRT will increase substantially [32].

The profile of the malnutrition—hematology—inflammation complex has not been systematically studied in juvenile Guatemalan ESRD patients, despite the existence of factors both intrinsic to the disease and extrinsic in the environment that bear on this triad. Our study had the objective to investigate the impact of ESRD on growth, anemia and/or inflammation, and to compare the findings to data reported elsewhere in order to describe the impact of background conditions in Guatemala. Differences along these parameters between HD and PD in the FUNDANIER clinic population are assessed and discussed as well.

## Subjects and Methods

### Subjects

Subjects were patients with confirmed ESRD assigned to one of two modalities of extracorporeal dialysis at the FUNDANIER renal clinic in Guatemala City, with the prospective notion to enroll equivalent samples for both modalities. At the time of the study, there were 27 children receiving HD thrice-weekly, of which all were enrolled. We recruited 27 children performing Continuous Ambulatory PD (CAPD) in the program for measurements and sample collection. The study was approved by the Human Studies Committee of the Center for Studies of Sensory Impairment, Aging and Metabolism (CeSSIAM) and the authorities of the Pediatrics Department of the Roosevelt Hospital, in which FUNDANIER operates. The families of the subjects agreed to participate voluntarily and signed an informed consent form about the benefits, potential risks and confidentiality. Each subject was allocated with a code number for identification. This study was registered at clinicaltrials.gov as NCT02369237.

Of the 27 HD patients, the cause of their chronic kidney disease was systemic lupus erythematosus nephritis in one instance, and congenital anomalies of the kidney and urinary tract in three others, while the remaining patients had idiopathic renal disease (n = 18) or there was no data available from an etiological work-up (n = 5). Across the PD subsample, one subject each had branchio-oto-renal syndrome and congenital anomalies of the kidney and urinary tract, while the idiopathic origin and non-evaluated categories represented 77.8% and 14.8% children, respectively.

Patients on HD received subcutaneous doses of 2,000 IU of EPO twice weekly and intravenous iron (iron sucrose, 100 mg/5 mL) during dialysis sessions, aiming to maintain a monthly control of ferritin levels between 500 and 1,000 ng/mL.

The membrane of the HD filter used is tricellulose acetate. This is not a high flux membrane. The machines are set to have a dialysis flow of 300 mL/min. The blood flow will depend on the size of the patient and the vascular access, but runs between 150–200 mL/min. Most of the patients have temporal catheters as vascular access. The ultrafiltration varies according to the weight gained between HD sessions (on average is 10–15% of the dry weight).

Although the health-care system of Guatemala cannot subsidize the purchase of erythropoiesis stimulating agents (ESA), erythropoietin analogs are available in the nation. Our clinic prescribes ESA for the patients as a subcutaneous dose 2000 IU of EPO once or twice a week; we estimate that about 70% of the families can provide some or all of the indicated dosage. In addition, oral iron was provided to patients on CAPD at a dosage calculated as from 2 to 6 mg/kg/day, administered on a twice-weekly basis.

### Anthropometric Measurements

All subjects had their weight (digital Health-o-Meter^®^ Professional) and height measured (SECA GmbH). HD patients were weighted before and after dialysis. Z-scores for common anthropometric indices were calculated using the WHO Global Infobase [33].

### Blood Extraction at Handling

At the end of a dialysis session, 8 mL of venous blood was drawn from blood tubing in HD patients and 8 mL by percutaneous venipuncture in PD patients at their regular out-patient clinical visit. Five mL were kept in a tube containing EDTA as whole blood for hematological assessments. 1 mL was kept in plastic tubes, one designated for ferritin and CRP determination in a local laboratory, another sub-aliquot was preserved in a cryogenic tube to determine inflammatory biomarkers and most iron status assays in Austria; the remaining serum supernatant was placed in a cryogenic tube for hepcidin analyses in the Netherlands. The cryogenic tubes were stored at -80°C until shipment on dry-ice to the European collaborators.

### Hematological Indices

Within 4 h of extraction, complete hematology was performed in the EDTA treated samples at the routine laboratory of the Nuestra Señora del Pilar Hospital in an automated hematological profile analyzer (Cell-Ruby, Ruby™, Abbott Diagnostics, Santa Barbara, Calif., USA). The hemogram information analyzed for this study included hemoglobin (Hb) concentration, expressed as g/dL. The Hb target for treating anemia in FUNDANIER is 12 g/dL. According to Dirren et al, 1994 [34], the criterion for anemia for the altitude of Guatemala City (1,500 m) should be adjusted by +0.7 g/dL in order to account for the lower oxygen tension. Therefore, the adjusted pediatric hemoglobin (Hb) cut-off criteria for anemia at sea-level were 12.2 g/dL for children between 5 and 12 years, and 12.7 g/dL for older subjects [34,35]. We also recorded the hematocrit (Htc) for the adjustment of erythrocyte sedimentation rate (ESR).

### Iron status Indices

Indicators of iron status, with the exception of soluble transferrin receptor (sTfR), were measured in Guatemala. Ferritin (reference range: 20–250 ng/mL) was determined by use of an ARCHITECT i1000 (Abbott Diagnostics) at the Guatemalan hospital laboratory. Serum iron (50–120 μg/dL), total iron-binding capacity (TIBC) (240–450 μg/dL) and transferrin (200–360 ng/mL) were measured photometrically; the ranges of the corresponding normal values for the respective age groups are given in brackets. sTfR (1.9–5.0 mg/L) was determined in Innsbruck by use of a Roche Hitachi 912 Chemistry Analyzer (Roche Diagnostics GmbH, Germany).

### Inflammation Indices

White blood cell count (WBC) from the aforementioned clinical hemogram, was expressed in cells per mm^3^. CRP was measured by nefelometry (Minineph Modelo AD500; < 5 mg/L); both were quantified at the clinical laboratory in Guatemala City. Erythrocyte sedimentation rates (ESR) (< 20 mm/h) were determined in Westergren vessels and adjusted for Htc. IL-6 (4.7–300 pg/mL), IL-10 (7.8–500 pg/mL), and TNF-α (4.7–300 pg/mL) were determined in Innsbruck (Quantikine^®^ Immunoassay; R&D-Systems, Wiesbaden, Germany).

### Determination of Hepcidin-25

Serum Hep-25 was measured in the Netherlands by a combination of weak cation exchange chromatography and time-of-flight mass spectrometry (WCX-TOF MS) [36]. An internal standard (synthetic heavy Hep-25 stable isotope +40; custom made Peptide International Inc.) was used for quantification [37]. Peptide spectra were generated on a Microflex LT matrix-enhanced laser desorption/ionisation TOF MS platform (Bruker Daltonics, Bremen, Germany). The lower detection limit of this method was 0.5 nM; average coefficients of variation were 2.8% (intra-run) and 6.4% (inter-run) [37]. The median reference level of serum Hep-25 in adults is 4.5 nM for men (reference range <0.5–<14.7 nM), 2.0 nM for premenopausal women (<0.5–<12.3 nM), and 4.9 nM (<0.5–15.6 nM) for postmenopausal women. These reference levels for the WCX-TOF MS method are derived from those of a previously published ELISA method, based on the regression line between the ELISA and WCX-TOF MS results obtained for the same samples from patients without hepcidin isoforms [36,38].

### Data Handling and Statistical Analyses

All values were keyed into Microsoft Excel Professional Academic 2010 for Windows. If data were normally distributed, values are given as the arithmetic mean ± SD. If not normal in distribution, log transformation was performed on the data, and we provided the median along with the 25^th^ and 75^th^ percentile. Nutritional status was assessed by calculating z-scores for height-for-age (HAZ) of each child by means of the WHO AnthroPlus Software v.1.0.2 (2007). Correlations were calculated according to Pearson. Linear regression analysis was carried out to assess the relationship between serum hepcidin and other parameters. Results were compared between HD and PD patients by the Mann-Whitney-Rank Sum Test for non-normally distributed parametric data, as parameters in populations with different age, degrees of renal failure and collateral inflammatory and hematological impairments cannot be considered normally distributed. Statistical analysis was performed using SigmaPlot^®^, Version 11.0 (2008, Systat Software Inc, San Jose, USA).

## Results

### Demographic and general characteristics

The characteristics of the subjects are presented in Table 1. Fifty-four children were recruited in FUNDANIER, Guatemala City. Twenty-seven of these children (13 females and 14 males) had been on HD treatments for 3 weeks to 42 months, whereas another 27 (16 females and 11 males) had been on PD therapy for periods from 1 to 58 months with the average time on dialysis being significantly longer for PD (p < 0.001). The ages ranged between 7 and 20 yrs; there was no significant difference in the mean age, height or weight between HD and PD children. The weight of HD children was measured after dialysis.

**Table 1:** Characteristics of the subjects.

	HD (n = 27)	PD (n = 27)	*p*-value*
Male [%]	52	41	0.29
Age [years]	12 (± 2) [7–16]	13 (± 3) [7–20]	0.49
Weight [Kg]	27 (± 8) [14–50]†	29 (± 7) [17–42]	0.35
Height [cm]	130 (± 14) [96–160]	134 (± 14) [110–157]	0.43
Height-for-age [z-scores]	-3.1 (± 1.3) [-5.9–-0.1]	-2.6 (± 1.4) [-5.7–-0.4]	0.16
Time on current dialysis [mo]	3.0 (95% CI: 2.6; 9.4) [0.75–42]	9.0 (95% CI: 8.5; 15.7) [1–32]	0.004
Total time on dialysis [mo]	4.0 (95% CI: 4.1; 14.0) [0.75–45]	14.0 (95% CI: 9.8; 20.4) [1–58]	0.031

HD, hemodialysis; PD, peritoneal dialysis; mo, months.

Data are means (± SD), median (95% Confidence Intervals) and [range].

*P values determined according to t-test for normally distributed continuous variables, Mann-Whitney rank-sum tests for non-normally distributed continuous variables.

^†^Weight measured after hemodialysis.

### Anthropometric classification

**Fig 1:**
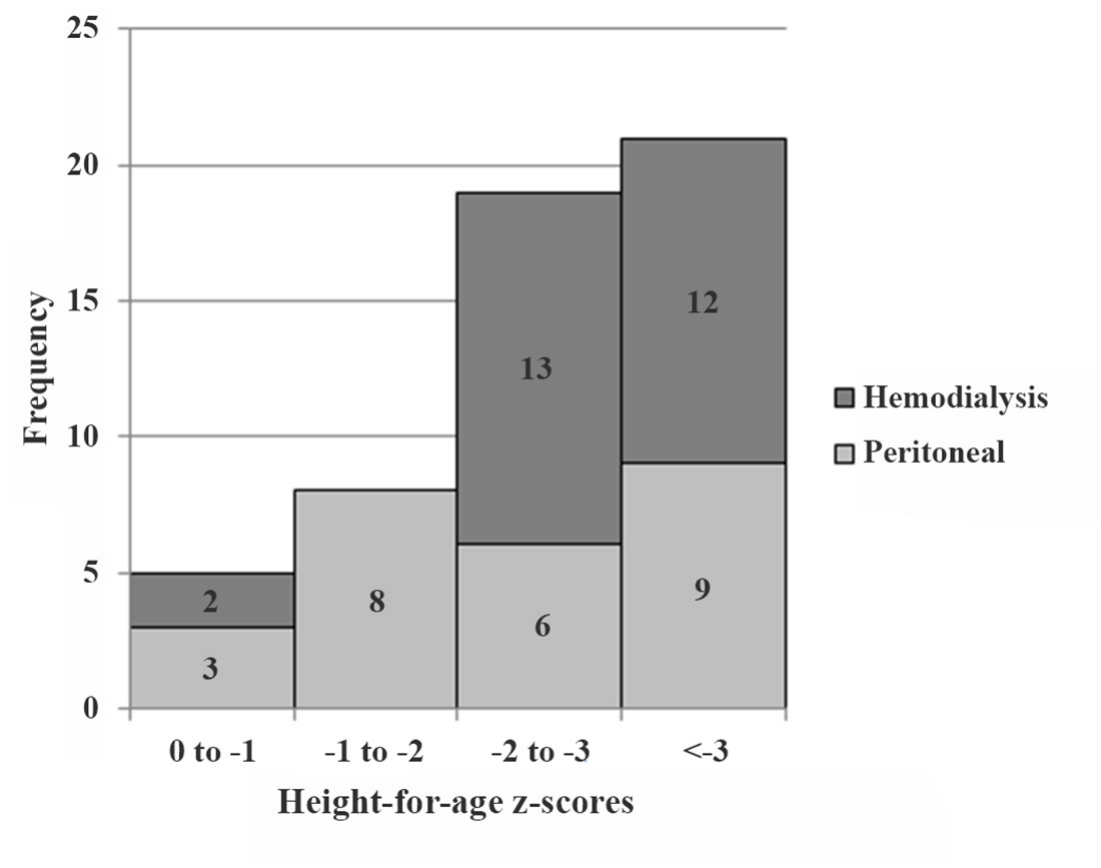
Histogram. Distribution of HAZ (n = 53). Grey bars represent peritoneal dialysis patients (n = 26) and dark grey bars represent hemodialysis patients (n = 27). Z-scores values between 0 and -1 = normal; between -1 and -2 = stunting; between -2 and -3, moderate stunting; and < -3.1 = severe stunting.

In reference classificatory terms, children in both subgroups were short. For the HD group heights ranged from 96 to 160 cm (mean: 130 ± 14 cm, median: 126 cm). Correspondingly, the range of height-for-age (HAZ) z-scores varied from -5.95 to -0.08. According to the WHO 2006 classification [33], 12 children (44.5%) were severely stunted (< -3.0 HAZ), 13 (48%) were moderately stunted (z-score: < -2.0 to -3.0 HAZ), and 2 (7.5%) were not stunted (> -2 HAZ). Corresponding heights for the PD group ranged from 110–157 cm (mean: 134 ± 13 cm, median: 138 cm). The range of height-for-age (HAZ) z-scores varied from -5.73 to -0.37. 33% of the children were severely stunted, 22% were moderately stunted, and 45% were not stunted (Fig 1). Chi-square analysis with Yates correction showed a significant difference of the proportion of children in stunted categories in the HD compared to the PD subgroups (*P* = 0.005).

### Hematological indices

**Fig 2:**
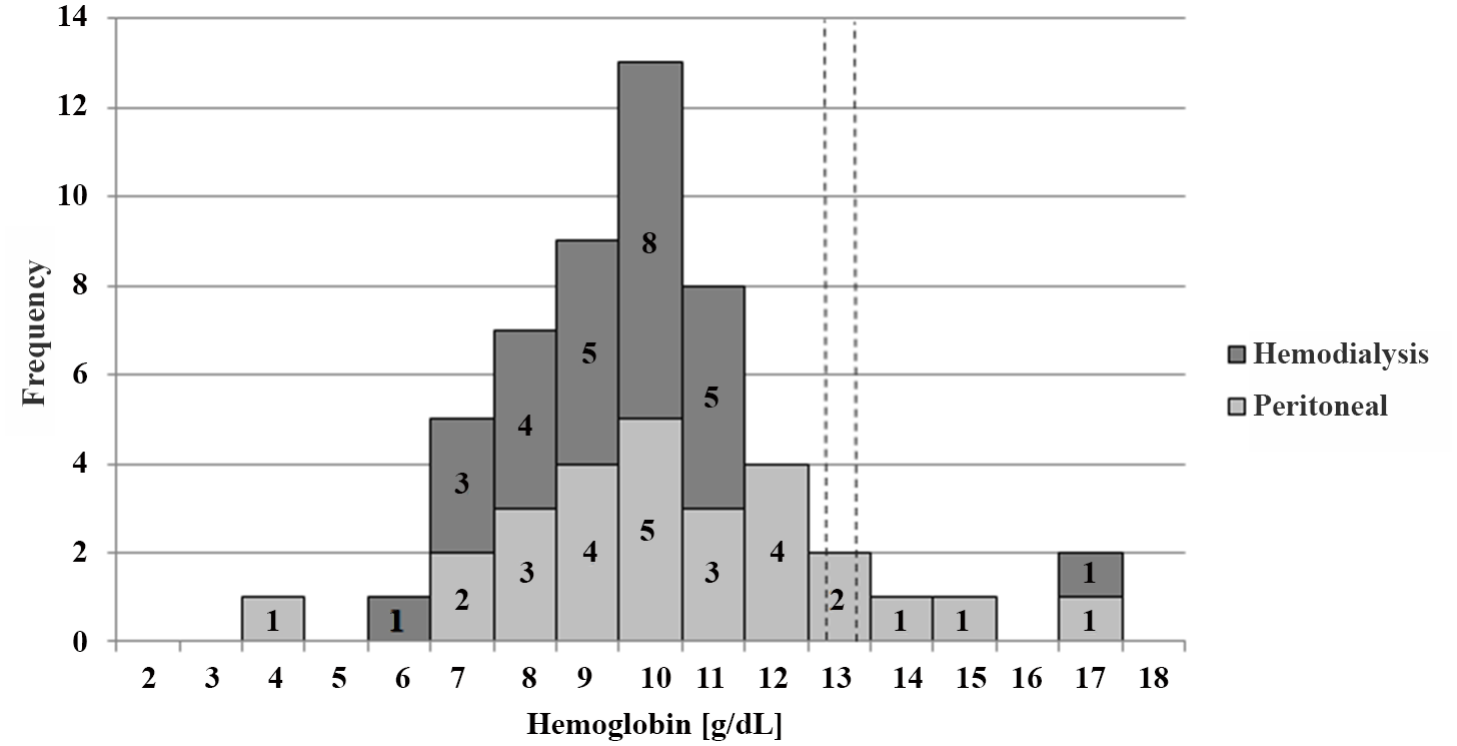
Histogram. Distribution of whole blood hemoglobin concentrations in g/dL of all patients (n = 54). Grey bars represent peritoneal dialysis patients (n = 27) and dark grey bars represent hemodialysis patients (n = 27). Vertical dashed lines show the cut-off values for hemoglobin in Guatemala for children between 5 to 12 years of age (12.2 g/dL) and children between 12 and 15 years of age (12.7 g/dL).

Hematological measured parameters for iron and inflammatory biomarkers are presented in Tables 2 and 3. Hb mean, SD and range values are shown in Table 2. Median Hb values were 9.3 and 9.0 g/dL for PD and HD patients respectively. Correspondingly, prevalence of anemia is shown in Table 3. The distribution of these values is depicted in Fig 2. The altitude-adjusted criteria for children were applied individually to the assessment of anemia [34], identifying 85% of the PD and 96% of the HD subjects as being anemic.

**Table 2:** Central tendency and variance values for iron status and inflammatory biomarkers in hemodialysis and peritoneal dialysis patients.

	HD (n = 27)	PD (n = 27)	p-value*
**Iron Status Biomarker**			
Hb [g/dL]	9.0 (± 2.0) [3.8–16.5]	9.9 (± 2.7) [6.0–16.5]	0.17
Serum iron [μg/dL]†	65 [24–148]	65 [30–175]	0.29
TIBC [μg/dL]	282 [143–488]	253 [154–340]	0.53
Transferrin [ng/mL]†	21 [7–63]	28 [12–103]	0.23
sTfR [mg/dL]	3.4 [1.8–6.1]	2.7 [0.3–7.1]	0.14
Ferritin [ng/ML]†	602 [193–4,113]	458 [17–1,506]	0.01
Hep-25 [nM]	36 [2–131]	43 [0.5–140]	0.68
**Inflammatory Biomarker**			
WBC [10^3^/μL]	5.2 [2.7–14.1]	6 [4–12]	0.04
ESR [mm/h]	97 [16–145]	75 [2–125]	0.03
CRP [mg/L]	[< 5–70] ǂ	[< 5–62] ǂ	NA
IL-6 [pg/mL]	7 [3–335]	3 [1–13]	< 0.001
IL-10 [pg/mL]	14 [5–690]	6 [2.5–65]	< 0.001
TNF-α [pg/mL]	6.5 [0–122]	[0–9] ǂ	< 0.001

HD, hemodialysis; PD, peritoneal dialysis; Hb, hemoglobin; TIBC, total iron binding capacity; sTfR, soluble transferrin receptor; Hep-25; hepcidin-25; WBC, white blood cell count; ESR, erythrocyte sedimentation rate; CRP, C-reactive protein; IL-6, Interleukin-6; IL-10, Interleukin-10; TNF-α, tumor necrosis factor alpha; NA, not available.

Data are presented as means (± SD), or median and [minimum-maximum].

* P values determined according to t-test for normally distributed continuous variables, Mann-Whitney rank-sum tests for non-normally distributed continuous variables.

^†^ Log transformed variables.

^ǂ^ Only presented the range.

**Table 3:** Percentage of subjects with values outside of the normal range for biomarkers in hemodialysis and peritoneal dialysis patients.

Percentage abnormal (%)			
	HD (n = 27)	PD (n = 27)	*p*-value*
**Iron Status Biomarker**			
Hb [g/dL]	96.3	81.5	0.35
Serum iron [μg/dL]	33.3	37.0	1.0
TIBC [μg/dL]	55.5	22.2	0.02
Transferrin [ng/mL]	51.8	44.4	0.79
Transferrin saturation [%]	59.2	37.0	0.17
sTfR [mg/dL]	11.1	33.3	0.10
Ferritin [ng/mL]	92.6	63.0	0.02
Hep-25 [nM]	85.2	81.5	1.0
**Inflammatory Biomarker**			
WBC [10^3^/μL]	29.6	11.1	0.17
CRP [mg/L]	25.9	3.7	0.05
ESR [mm/h]	88.8	92.6	1.0
IL-6 [pg/mL]	40.7	3.7	0.23
IL-10 [pg/mL]	25.9	14.8	0.5
TNF-α [pg/mL]	11.1	0	0.002

HD, hemodialysis; PD, peritoneal dialysis; Hb, hemoglobin; TIBC, total iron binding capacity; sTfR, soluble transferrin receptor; Hep-25, hepcidin-25; WBC, white blood cell count; CRP, C-reactive protein; ESR, erythrocyte sedimentation rate; IL-6, Interleukin-6; IL-10, Interleukin-10; TNF-α = tumor necrosis factor alpha.

* P-value was calculated with original data.

**Fig 3:**
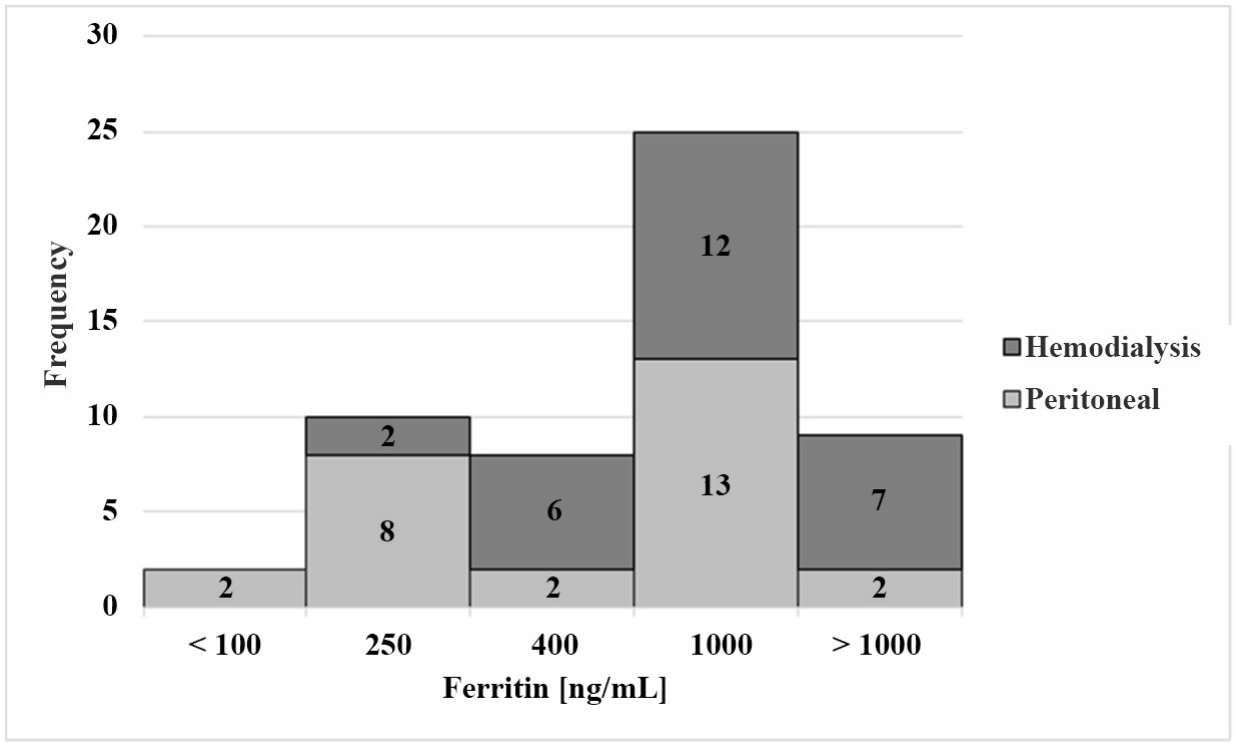
Histogram. Distribution of ferritin concentrations in ng/mL of all patients (n = 54). Grey bars represent peritoneal dialysis patients (n = 27) and dark grey bars represent hemodialysis patients (n = 27).

Laboratory biomarkers to assess the iron status are shown in the upper panels of Tables 2 and 3. The only significant differences between both treated groups were found for ferritin, with higher increments in the HD subgroup. This distribution is illustrated in the histogram of Fig 3. Of special note were the upper ranges of serum ferritin concentrations, with the extreme value in the HD group exceeding 4,000 ng/mL and the highest values for PD patients exceeding 1,500 ng/mL. Over 30% of values were outside of the normal cut-off boundaries for iron-status indicators with the exceptions of sTfR in the HD subgroup and of TIBC in the PD subgroup.

**Fig 4:**
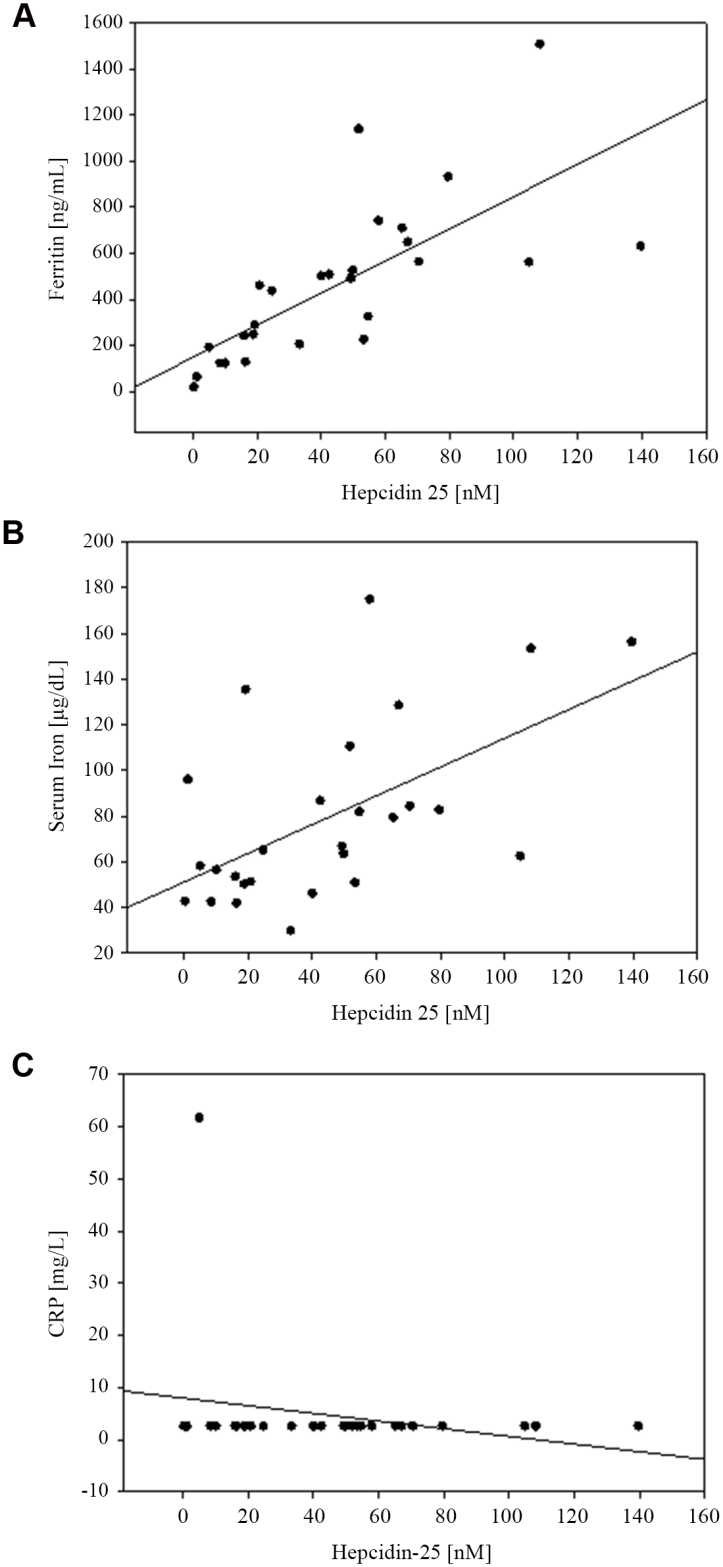
Scatter Plot. Correlation in PD patients between **A**. ferritin and Hep-25 (*r* = 0.77; *P* < 0.01); **B**. serum iron and Hep-25 (*r* = 0.56; *P* < 0.01); and **C**. CRP and Hep-25 (*r* = -0.23; *P* = 0.256).

**Fig 5:**
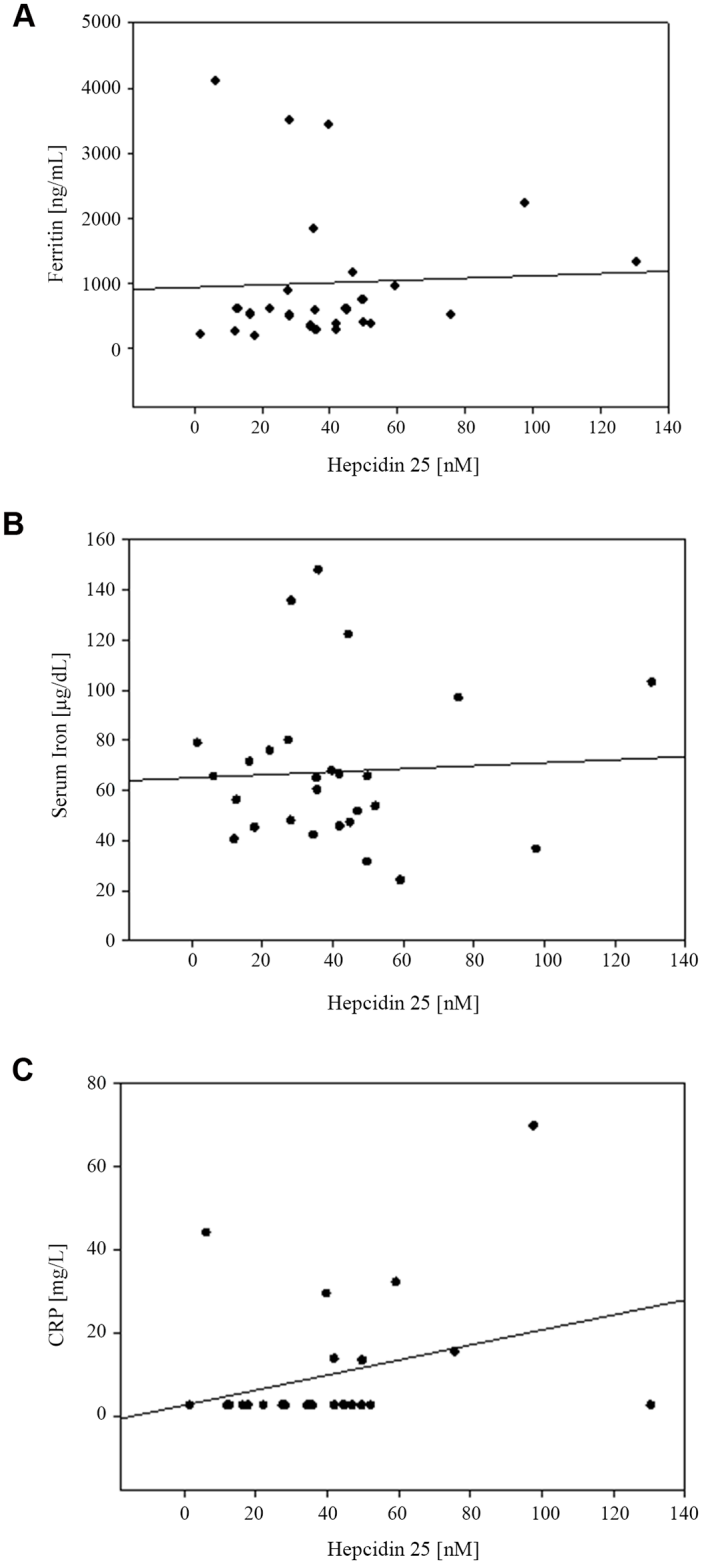
Scatter Plot. Correlation in HD patients between **A**. ferritin and Hep-25 (*r* = 0.04; *P* = 0.82); **B**. serum iron and Hep-25 (*r* = 0.05; *P* = 0.79); and **C**. CRP and Hep-25 (*r* = 0.31; *P* = 0.12).

Hep-25 concentrations ranged from 0.5 to 140 nM for PD and 2 to 131 nM for HD patients (p = 0.7). Comparing these concentrations to the normal range (< 0.5 to 14.6 nM), 81.5% of PD and 85.2% of HD patients had elevated Hep-25 concentrations (Table 3). Table 4 presents the correlation of Hep-25 with the iron and inflammatory variables (Spearman test). Significant positive correlations were observed between Hep-25 and serum iron-concentrations, transferrin and ferritin for PD. Whereas negative correlations were observed between Hep-25 and hemoglobin, TIBC and serum transferrin receptor in the same group. These correlations were not significant for the HD patients. Accordingly, Hep-25 correlated well with ferritin (r = 0.77; p < 0.01) and serum iron (r = 0.56; p < 0.01) in PD (Fig 4), but not in HD patients (Fig 5) when analyzed by the Pearson test.

**Table 4:** Spearman correlation coefficients of Hep-25 with other variables.

	HD (n = 27)		PD (n = 27)	
Variable	*r*	*p*-value	*r*	*p*-value
Age [years]	0.14	0.47	-0.2	0.23
Weight [Kg]	0.13	0.5	-0.2	0.29
Height [cm]	0.17	0.4	-0.4	0.03
Hb [g/dL]	0.08	0.68	-0.4	0.02
Serum iron [μg/dL]	-0.1	0.56	0.6	0.002
TIBC [μg/dL]	-0.2	0.31	-0.3	0.07
Transferrin [ng/mL]	0.06	0.7	0.6	< 0.001
sTfR [mg/dL]	-0.3	0.18	-0.6	< 0.001
Ferritin [ng/mL]	0.2	0.26	0.9	< 0.001
WBC [10^3^/μL]	0.06	0.77	0.2	0.2
CRP [mg/L]	0.3	0.09	-0.3	0.16
ESR [mm/h]	0.3	0.15	0.3	0.15
IL-6 [pg/mL]	0.14	0.47	0.1	0.44
IL-10 [pg/mL]	0.2	0.36	-0.1	0.51
TNF-α [pg/mL]	0.13	0.48	0.2	0.32

HD, hemodialysis; PD, peritoneal dialysis; Hb, hemoglobin; TIBC, total iron binding capacity; sTfR, soluble transferrin receptor; WBC, white blood cell count; CRP, C-reactive protein; IL-6, Interleukin-6; IL-10, Interleukin-10; ESR, erythrocyte sedimentation rate; TNF-α = tumor necrosis factor alpha.

### Inflammatory Biomarkers

Ferritin and hepcidin, discussed with the iron-status markers above, are also acute-phase reactants and are, thus, closely related to concentrations of inflammatory biomarkers shown in Tables 2 and 3.

**Fig 6:**
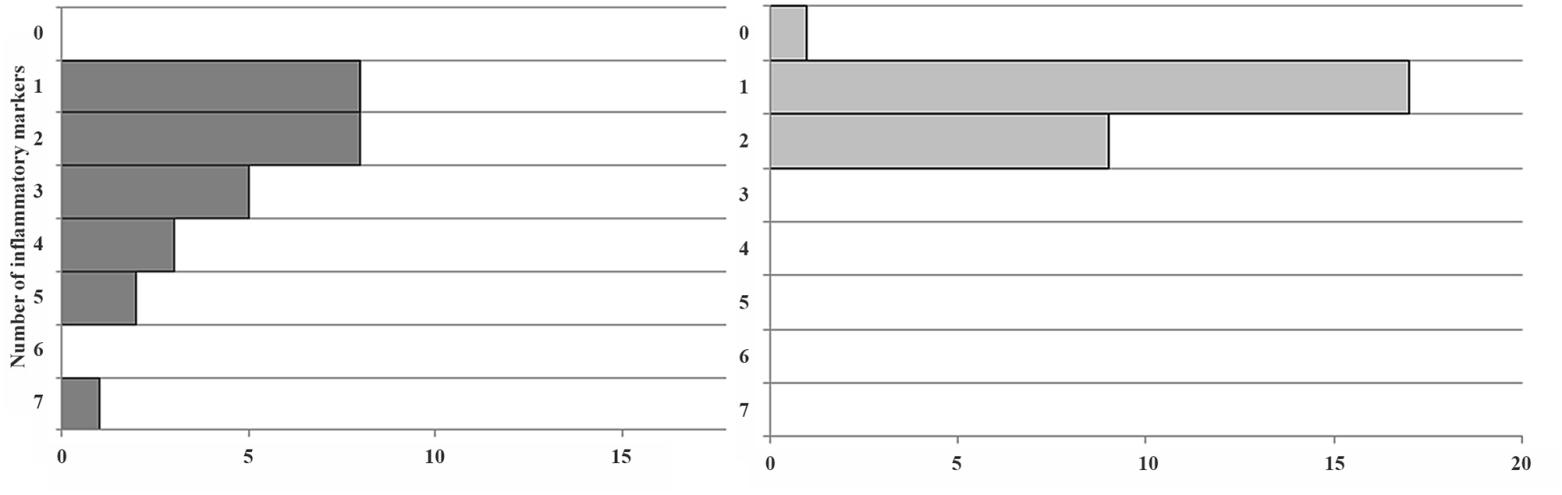
Frequency of concurrently elevated inflammatory biomarkers in the same HD and PD patients.

The percentage of abnormally elevated biomarkers was higher in numerical terms for HD in 5 out of 6 (as well as for ferritin and hepcidin), and this was significant for ferritin (p = 0.02) and TNF-α (p = 0.002 Chi-square). The only exception was the mild reversal for ESR, being 5 percentage-points more frequently elevated on the PD side. As shown in Fig 6, the clustering of concurrently elevated inflammatory biomarkers in the same patient shows a marked effect: whereas 100% of PD subjects had 2 or fewer elevations, 11 of 27 HD patients (41%) showed 3 or more parameters elevated. Statistical analysis is not appropriate, giving the nature of the distribution, but we get the visual impression that the cumulative burden of systemic inflammation is greater in the HD group.

## Discussion

End-stage renal disease (ESRD) in children presents a multi-level challenge for a developing country like Guatemala. The resources to support facilities for care and therapy in pediatric nephrology are severely limited. As discussed above, moreover, background factors in Guatemala involving diet, environment and economics can be argued to already provide a “head start” down the slope towards poor growth, anemia and inflammation, the cardinal manifestations of interest in this study. The background states for the three kinds of manifestation were postulated to interact with renal-based factors in an additive—if not synergistic—manner, to produce growth retardation, hematological deficits and more rigorous inflammatory response in the extreme end of what has been reported in the literature.

### Comparative issues of linear growth

The stunting rate in Guatemala of 49.8% for children under 5 years of age is the third highest reported worldwide [4]. For comparison, average stunting rates of 38%, 28% and 14% have been reported for Africa, Asia and Latin America, respectively [33]. Underlying causes under discussion range from preconceptual and prenatal factors, such as short stature of the mother, early age of first delivery, maternal underweight and anemia, to tobacco use and indoor pollution. Thus, the adolescents in our study could well have gone through a period of retarded growth during this vulnerable prenatal period. In addition, there are postnatal influences, such as inadequate nutrition and frequent infections [39]. Intergenerational transmission of stunting is mediated by shared genetic characteristics, inherited poverty, and by epigenetic influences [40–43]. Growth faltering begins soon after birth, reaching a maximum at 2 years of age after which the extent of stunting seems to remain at the same level [44,45]. Impaired school performance and increased morbidity and mortality are often associated with stunting. The prevalence and extent of stunting in Guatemalan ESRD juveniles are significantly higher than observed in a community trial in Guatemala City during the same period which argues for a predominant contribution of renal impairment to the stunting rates observed in the present study. Causes of stunting seem closely related to nutrition, as shown by the positive effect of nutritional interventions in Guatemala [46], and by improved height and body proportions in Mayan children who moved from Guatemala to the US [47].

Uremic growth failure is multifactorial, including causes like energy malnutrition, water and electrolyte disturbances, metabolic acidosis, renal anemia and somatotropic and gonadotropic hormone disturbances [48]. Half of the patients with ESRD in childhood attain adult’s height below the third centile [49]. Inflammation and impaired energy metabolism reduce appetite, which, in a vicious cycle, reduces the supply of vitamins C, B6, B12 and folate [50]. US data show that the extent of stunting does not respond to dialysis [51,52], while renal transplantation before or during the prepupertal growth period induces moderate catch-up growth [52]. Growth failure has long been recognized in children with chronic renal failure [53]. However, data on the prevalence of stunting in ESRD are scarce, particularly in developing countries. Approximately 50% of children who develop ESRD in Europe before the age of 15 were stunted [54], which compares to 93% stunted juveniles in our cohort (45% severe plus 48% moderate stunting). These figures suggest a much higher prevalence of stunting in Guatemalan than in European ESRD patients. The comparison remains incomplete though, as we do not have sufficient access to the quoted European database to compare the different degrees of stunting. Average HAZ scores in US ESRD children varied between -1.81 ± 0.05 and -2.18 ± 0.12 [51], which again signals considerably less stunting than the average of -3.1 ± 1.3 and -2.6 ± 1.4 found in Guatemalan children on HD and PD, respectively.

Bone growth is the most essential determination of height, requiring the optimal conditions for elongation to achieve genetic potential in stature. Skeletal elongation is largely a hormonally-mediated process, but the mineralization disruption of renal osteodystrophy, discussed in the context of anemia [55,56], could limit the efficacy of the trophic hormone signaling due to the limitation of mineral substrates for new bone formation.

### Comparative issues of hematological status

The average prevalence of anemia in Guatemalan children is 38% [5,57]. About half of the anemia cases in developing countries go along with low ferritin values indicating iron deficiency anemia (IDA) [58,59]. High dietary phytate and polyphenol content which impair iron bioavailability have been discussed as underlying causes, in combination with low intake rates of meat, fish, poultry, and fruits rich in ascorbic- and polyoxicarbonic acids to counteract this impairment [60]. Anemia in combination with high ferritin concentrations has been termed non-IDA and accounts for the other half of anemias in developing countries. These anemias were discussed to relate to lack of essential micronutrients other than iron, such as vitamins A, B2, B12, C or Cu, or Zn [58] or to inflammation.”Anemia of inflammation”, also termed “Anemia of chronic diseases” (ACD), goes along with increased hepcidin concentrations that reduce duodenal iron absorption and increase iron sequestration in the reticuloendothelial system. Both effects are supposed to serve as host-defense mechanisms in infection, but restrict iron procurement to erythropoiesis in parallel [26].

Anemia in ESRD is regarded to have traits of ACD showing increased IL-6 and hepcidin concentrations, so that iron is rendered scarce for erythropoiesis. This may act additively with the nutrition-dependent mechanisms of anemia in developing countries described above. Glomerular hepcidin filtration is impaired in renal failure, which contributes to increased hepcidin concentrations in ACD. Consequently, hepcidin can be successfully reduced by HD [61–64]. However, post-dialysis levels were back to pre-dialysis levels within one hour after the end of the dialysis session [65]. Another major cause of anemia in ESRD is EPO-deficiency [66]. Still, EPO administration fails to reduce anemia in 10% of patients. It has been speculated that Hep-25 concentrations in serum might predict the response to EPO administration [66]. However, in humans the association between hepcidin and erythropoiesis stimulating agents (ESA) response has not been definitely demonstrated [67]. Moreover, hepcidin seems to inhibit erythropoietic colony formation directly [68] and erythrocyte survival is shortened due to hemolysis in patients with ESRD, likely also in the dialysis circuit [69,70]. In addition, sufficient iron procurement was shown to be helpful in the treatment of anemia in ESRD [71].

A potential contributor to anemia could be the renal osteodystrophy seen in ESRD patients, in which the elevation of circulating phosphate produces decreased levels of calcium and subsequent elevated parathyroid hormone (PTH) [72]. A prominent feature of this condition is fibrosis in the bone marrow, which crowds out the medullar space for the development of red cell precursors. Controlling the consequences of accumulation of phosphorus is the therapeutic strategy to avert its leaching of bone calcium. Our patients are prescribed a daily dose of 0.25 μg of 1-*alpha* vitamin D3 in order to create a negative feedback to PTH, and with calcium carbonate tables to enhance exogenous calcium uptake and to chelate the phosphorus of the diet. Therapy is gauged by PTH maintained at least at three times the 75 pg/mL basal concentration. In analyzing the respective assay data available at the time of study across the 54 patients, only 4 of 19 PTH values failed to exceed the 225 pg/mL threshold. Hypocalcemia (< 8.0 mg/dL) was only seen twice among 16 assays, but hyperphosphatemia (>5.0 mg.dL) was present in 45% if the 22 values reviewed (data not shown). This information opens the way for fibrotic issues of the bone marrow to be affecting red cell production in some individuals.

Independent of the underlying causes, recent trials [73] signal a higher frequency of fatal and non-fatal cardiac events in individuals with normal as compared to reduced hemoglobin and hematocrit concentrations; this casts doubt on whether reducing prevalence of anemia should be a primary goal of treatment in ESRD, although it is prudent to support Hb concentrations at a certain minimal level, even within the anemic range.

### Iron status regulation and inflammatory burden

Hepcidin measurements are not standardized and, therefore, absolute hepcidin levels cannot be reliably compared if obtained by different methods [74]. Adult patients with ESRD from different parts of the world often present with small to massive hepcidin elevations [62–64,75]. In a study from California, USA, using an immunoassay for serum hepcidin, the median values of hepcidin were 26 times higher in juvenile hemodialysis patients (average age of 15 y) than those in age-equivalent control children with normal renal function [76]. In adolescents and adults on hemodialysis in California, USA [63], the adults had a three-fold higher hepcidin concentration than juveniles, which was observed in control as well as in HD subjects. In contrast, with the WCX-TOF-MS method from Nijmegen, the relationship Dutch adults on hemodialysis [64] and children of both groups in this study was reversed. The median hepcidin value of 9.4 nM in adults was one-fourth that in Guatemalan juveniles on HD and one-fifth that in those on PD. Two differences between these situations may be responsible for this inversion. Firstly, lower hygienic status and frequent exposure to infectious microbes as well as noise and pollution may increase stress and the inflammatory burden in Guatemala. The latter should increase biomarkers such as CRP, leukocytes, and cytokines in Guatemalan children which, in turn, is likely to raise hepcidin concentrations in parallel, aggravate anemia and impair prognosis of children with ESRD. CRP concentrations, for instance, were much higher in Guatemalan juveniles than in Dutch adults on HD (26 mg/L vs. 8 mg/L). Secondly, parenteral iron dosing in Guatemalan children on HD lead to an average ferritin level of 600 ng/mL while Dutch adults showed a median ferritin concentration of 270 ng/mL. Guatemalan patients did not receive any blood transfusion, which would explain the iron overload in this population. Inflammatory burden and iron supplementation differ markedly between these two groups which can well overrule age-related differences.

### Correlation between plasma concentrations of Hep-25 and ferritin in ESRD

In Dutch adults using the same WCX-TOF MS Hep-25 assay [64] as in the present study, those patients with chronic kidney disease not yet on dialysis showed a high correlation coefficient (r = 0.74) between ferritin and hepcidin concentrations. Those patients on HD for a median period of 26 mo, had an r = 0.79. This is in accordance to the expectation, as inflammation and high iron status stimulate hepcidin synthesis in parallel [67,77]. Focusing on the hemodialysis patients in the Dutch study, significant correlations were also detected between Hep-25 and Hb, serum iron, transferrin and CRP, but not with transferrin saturation. In contrast, in the present study of juvenile Guatemalans on HD, no significance was found for the association of any of the aforementioned variables (Table 4). However, in the PD subgroup, we replicated significant correlations with all common variables mentioned above, except CRP (Table 4). Scattergrams of associations between Hep-25, ferritin and serum iron are shown in the respective panels of Fig 6. In our PD group, moreover, the association was also significant with sTfR as well as with height in cm (Table 4).

One possible explanation for these differences is, again, the differential use of parenteral iron supplementation. Our PD patients received oral iron. In the study by Peters et al [64], adult subjects received no i.v. iron during hemodialysis, resulting in a median ferritin concentration of ~270 ng/mL. Our HD patients were given parenteral iron during dialysis sessions, aiming to maintain ferritin levels between 500 and 1000 ng/mL and succeeding to establish a median ferritin concentration of ~600 ng/mL (Table 2). These values are over twice as high as those observed in Dutch adults [64] and suggest liver iron overload [78,79]. In the Guatemalan PD children iron was given orally. Thus, the extent of its absorption is under hepcidin control and will be reduced in inflammation and with high iron status. We speculate that the lesser exposure to iron in our PD series and in the Dutch adults produced a situation in which ferritin was faithfully reflecting iron stores, and hepcidin was responding to regulatory needs. The higher iron doses may have led to a situation in which both the iron-storage and the low-grade inflammation indicated by the corresponding inflammatory biomarkers have an impact on circulating ferritin values. This is compatible with the obviously higher inflammatory burden in the HD patients shown in Fig 5. Further research is needed to determine if the administration of iron preparations to ESRD patients produces adverse effects and aggravates other comorbidities in the patients (viral hepatitis, cardiovascular disease, infections).

### Interplay between inflammatory burden, stunting and anemia

In children on HD the high number of increased inflammatory markers indicates a higher level of inflammation than in PD. Transgenic mice with markedly increased IL-6 levels show a negative correlation between IL-6 and insulin-like growth factor I (ILGF I) and are stunted [80]. This may be one of the mechanisms by which inflammation reduces growth. Correspondingly, TNF-α concentration correlated with the degree of stunting in girls, and IL-6, CRP and insulin resistance correlated with the degree of stunting and adiposity in boys in South Africa [81]. This corresponds well to the higher degree of stunting in the HD as compared to PD juveniles of our study with less indication for inflammation (Figs 1 and 4). Higher stunting rates in HD children may also explain why hemoglobin concentrations showed no differences between the treatment groups in spite of markedly higher hepcidin values in the HD children. Blood volume is assumed to account for approximately 3% of body weight. Thus, smaller children have a lower blood volume and the same amount of hemoglobin will lead to a higher hemoglobin concentration. This corresponds, at least in part, to Golden’s hypothesis of stunting as an adaption to micronutrient deficiency [82].

One of the factors for a greater burden of inflammatory stress in the HD group, as compared to the PD group, could theoretically have related to the cumulative duration of renal replacement therapy. However, the data do not support such a proposition (Table 1). In fact, paradoxically, the PD patients had been receiving RRT for over three times as long (14 mo) as those on HD (4 mo), such that duration, per se, is not a viable candidate factor for determining inflammatory intensity. With respect to peritonitis, this would be a candidate factor contrary to that found in this study, namely one of a greater inflammatory state in the PD series. As expected, some HD children have been on PD earlier; records showed that of the seven patients on HD who had previously undergone PD, two had a history of peritonitis episodes. Only 10 of the 27 patients of the study currently on PD were free of a peritonitis history, with the incidence of episodes ranging from 1 to 32 in the remaining subjects. Finally, vascular access for the HD series is a candidate factor in differential inflammation. In Guatemala, hemodialysis does not come with the luxury of permanent arterio-venous fistulas and temporary catheters must be used. This may represent a partial explanation for enhanced inflammatory stress in the HD group.

### Exceptional behavior of hemoglobin, ferritin and hepcidin concentrations

Finally, we can take a closer look into the subset of 4 ESRD patients on PD who did not have anemia, as shown in the hemoglobin distribution histogram in Fig 2. These hemoglobin values are in the right hand shoulder of the distribution curve above 12.7 g hb/dL, along with the one non-anemic patient from the HD group. These patients had low hepcidin values, diverging from the almost generalized high-hepcidin response in all other subjects in both dialysis modes. Similarly, the ferritin values were < 400 ng/mL in all non-anemic PD subjects (16.5; 61.6; 120.7; and 322.6 ng/mL). Obviously, the iron-transport blockade, mediated by hepcidin, is not operating in these 4 exceptional subjects, allowing their bone marrows to take up iron and effectively utilize it for erythropoiesis which avoids anemia. The extents of renal failure as well as the time on dialysis are in the same order of magnitude as in the anemic patients. Why the vicious cycle of renal failure and anemia is not operating in these patients requires future research.

### Strengths and limitations of the study

Among the strengths of the study is the comparably large scope of collected data, consisting of 8 hematological variables, specific measurement of Hep-25 by mass spectrometry among them, and 6 inflammatory variables. Moreover, they are all collected in a total of 54 children from a developing country; this number of patients on dialysis is in the same order of magnitude as in the study of Peters et al [64]. As FUNDANIER is the only center for pediatric dialysis in Guatemala, this provides an assessment of close to all children on dialysis in this country at the point in time of the trial. This relates to the disadvantage that our patients were not randomly allocated to HD or PD treatment, but for the best of the patients as for as achievable by available means. The different modes of iron treatment in the PD and HD group is another disadvantage, as it impairs direct comparability of the two treatment groups.

## Conclusions and Perspectives

Though to reduce extent and prevalence of anemia improves well-being, it seems to impair rather than to improve the patients’ life expectancy [73]. An expected finding was the much higher hepcidin and ferritin concentration in Guatemalan children as compared to Dutch adults. Future research should probe the underlying causes which may be found in the higher iron procurement and/or in the higher inflammatory burden of the Guatemalan children likely as a consequence of repeated exposure to infectious hazards. The impact of different iron dosages and different ways of iron administration in PD and HD patients impairs the direct comparison between the treatments, though it reflects presently used treatment strategies. Finally, 4 PD children showed no anemia and 3 of them showed no increased hepcidin or ferritin values; this triad of findings in itself fits nicely together. However, anemia, iron metabolism and inflammation in those 4 patients behave differently from the majority of the children with ESRD in this study. Speculation on the causes underlying this less dramatic clinical course may be differences in iron procurement, in the extent of inflammation or in genetic aberration of second messenger pathways of hepcidin regulation. It seems promising to investigate such “black swan” events in more detail when looking for new modes of treatment for children on ESRD.

## Abbreviations

ACD: anemia of chronic disease
BMI: body mass index
CAPD: Continuous Ambulatory Peritoneal Dialysis
CRP: C-reactive protein
EAS: erythropoiesis stimulating agents
EDTA: ethylenediaminetetraacetic acid
ELISA: enzyme linked immunosorbent assay
EPO: erythropoietin
ESR: erythrocyte sedimentation rate
ESRD: end-stage renal disease
FUNDANIER: Foundation for Children with Kidney Diseases
HAZ: height-for age z-score
Hb: hemoglobin
HD: hemodialysis
Htc: hematocrit
IDA: iron deficiency anemia
IL-6: Interleukin-6
IL-10: Interleukin-10
ILGF: insulin-like growth factor
IU: international units
PD: peritoneal dialysis
PTH: parathyroid hormone
RRT: renal replacement therapy
SD: standard deviation
sTfR: soluble transferrin receptor
TIBC: total iron binding capacity
TNF-α: tumor necrosis factor alpha
WBC: white blood cell count
WCX-TOF-MS: weak cation exchange chromatography time-of-flight mass spectrometry
